# Prevalence of Musculoskeletal Complaints in Underserved Populations Attending a Student-Run Free Clinic in Puerto Rico: A Cross-Sectional Analysis

**DOI:** 10.7759/cureus.108477

**Published:** 2026-05-08

**Authors:** Gonzalo F Del Rio Montesinos, Andrea Fabregas, Naishaliz Lorenzo Gonzalez, Claudia Baralt, Antonio Morales, Sofia Jimenez

**Affiliations:** 1 Orthopaedics, Universidad Central del Caribe, Bayamon, PRI; 2 Otolaryngology, Universidad Central del Caribe, Bayamon, PRI; 3 Anatomy, Universidad Central del Caribe, Bayamon, PRI

**Keywords:** chronic disease health, community health research, cross sectional studies, musculoskeletal disability, student-run clinic

## Abstract

Background: Musculoskeletal (MSK) disorders are a leading cause of disability worldwide and disproportionately affect individuals facing socioeconomic hardship. In Puerto Rico, recent social and economic disruptions have intensified health inequities, yet data on MSK burden in underserved populations remain limited. Student-run free clinics (SRFCs) provide a unique setting to characterize MSK health in these populations. This study aimed to determine the prevalence of musculoskeletal complaints and functional limitations among adults attending a student-run free clinic in Puerto Rico and to evaluate associated sociodemographic, occupational, and chronic disease-related factors.

Methods: We conducted a cross-sectional study among adults attending an SRFC in Old San Juan between February and December 2025. Participants completed a 30-item MSK Function Questionnaire assessing pain, functional limitations, strength, activity modification, and sociodemographic and health-related factors. Data were analyzed descriptively, and subgroup comparisons were performed using Fisher’s exact test.

Results: Sixty-two participants were included, most of whom were older, unemployed, and experiencing housing instability. Overall, 77.4% reported at least one MSK limitation. Lower extremity and spine-related symptoms were most common, with frequent reports of pain, functional difficulty, and activity modification. MSK limitations were significantly more prevalent among unemployed individuals and those with chronic medical conditions, particularly diabetes and cardiovascular disease. Multivariable logistic regression analysis demonstrated that chronic conditions and unemployment remained associated with MSK limitations after adjustment.

Conclusions: MSK pain and functional impairment were common among underserved patients attending an SRFC in Puerto Rico and were associated with chronic disease burden and unemployment. Given the small sample size, locally developed questionnaire, and cross-sectional design, these findings should be interpreted as preliminary and hypothesis-generating. The relationship between unemployment and MSK impairment should be interpreted as associative and potentially bidirectional.

## Introduction

Musculoskeletal (MSK) disorders are among the most prevalent causes of disability worldwide, affecting over 1.7 billion people and contributing substantially to years lived with disability across all regions [1]. These conditions, including arthritis, low back pain, and trauma‑related injuries, are major contributors to chronic pain, functional limitation, and economic burden. Despite their high prevalence, MSK complaints are often underrecognized in primary care settings, especially in low‑resource environments where competing health priorities dominate [1].

Articles in the medical literature discuss MSK health in underserved and unhoused populations from various perspectives. MSK disorders are highly prevalent and often more severe in unhoused and marginalized groups, with barriers to care leading to worse outcomes [1-7]. In low‑resource and underserved urban settings, MSK pain and arthritis are strongly associated with poor quality of life, functional impairment, and comorbidities such as hypertension and depression [2]. In high-income countries, unhoused individuals experience higher rates of unintentional injuries, falls, and chronic MSK pain, which frequently result in emergency department visits and hospitalizations [3]. Barriers to care, including lack of insurance, low health literacy, missed appointments, and limited access to specialty services, compound this burden [4,5,7]. Social determinants such as unemployment, housing instability, and mental illness further exacerbate morbidity and impede management [2,4,7,8]. Recent work emphasizes tailored community interventions, such as mobile medical units, to extend outreach and continuity of MSK care for populations experiencing homelessness [5,9].

These disparities are similarly reflected in Puerto Rico, where economic instability, provider migration, and natural disasters have intensified healthcare inequities. Adults on the island report high rates of chronic conditions, including arthritis, obesity, and painful inflammation closely linked to poor self‑rated health and limited access to consistent care [9]. The Puerto Rican population also faces disproportionate risks of frailty and disability associated with chronic MSK and metabolic conditions [10]. Nutritional and bone health studies among Puerto Rican adults show elevated rates of osteoporosis and low bone mass compared with other ethnic groups, underscoring their MSK vulnerability [11-13].

Student‑run free clinics (SRFCs) play a crucial role in bridging healthcare gaps for uninsured, unhoused, and underserved populations. These settings have the capacity to identify MSK conditions early, improve access to preventive care, and foster multidisciplinary training for future physicians. Yet, the prevalence and factors of MSK complaints within SRFC populations, particularly in Caribbean contexts, remain poorly characterized.

The primary objective of this study was to determine the prevalence of musculoskeletal complaints and functional limitations among adults attending a student-run free clinic in Puerto Rico. Secondary objectives included characterizing the anatomical distribution of MSK limitations and evaluating sociodemographic, occupational, behavioral, and chronic disease-related factors associated with self-reported MSK impairment.

## Materials and methods

Study design and setting

This cross-sectional observational study was conducted at Clinicas Padre Venard, a student-run free clinic operated by medical students at Universidad Central del Caribe in Old San Juan, Puerto Rico. Data collection occurred between February and December 2025. The study was approved by the Universidad Central del Caribe Institutional Review Board (IRB Protocol # 054-2025-17-06-IRB). All procedures were conducted in accordance with institutional and ethical standards.

Adult patients aged 21 years or older attending the clinic during the study period were invited to participate voluntarily. Exclusion criteria included age under 21 years, cognitive impairment precluding questionnaire completion, and refusal to participate. Participation was anonymous, and no identifiable information was collected. An IRB-approved waiver of written informed consent was granted; verbal consent was obtained prior to survey administration. 

Survey instrument and data collection

Participants completed a 30-item Orthopedic and Musculoskeletal (MSK) Function Questionnaire developed by the research team for this clinic-based study and adapted for local use. The questionnaire assessed multidimensional MSK health across six domains: (1) demographics, (2) upper extremity function, (3) lower extremity function, (4) spine and back function, (5) overall MSK function, and (6) general health and lifestyle factors. Items evaluated pain frequency, functional difficulty, self-perceived strength, activity modification, balance or coordination issues, and the impact of MSK symptoms on daily activities. The full Spanish and English versions of the questionnaire, including all items and response options, are provided in Appendix A.

Surveys were administered in either Spanish or English using REDCap and facilitated by trained medical student volunteers. Participants completed the questionnaire after their clinical visit in a private area to ensure confidentiality. Average completion time was approximately 15 to 20 minutes. All responses were anonymized and exported for analysis. 

Outcomes

The primary outcome was the presence of at least one self-reported MSK limitation in any body region (upper extremity [UE], lower extremity [LE], or spine/back). Participants were classified as having at least one MSK limitation if they reported difficulty or pain involving the upper extremity, lower extremity, or spine/back domains, or if they reported functional impact on daily activities. MSK limitation was therefore operationalized using questionnaire responses related to pain, difficulty with daily activities, activity modification, and regional functional impairment.

Additional variables included housing status, employment status, physical activity, sleep duration, and presence of chronic medical conditions. Chronic conditions were self-reported by participants through the questionnaire and categorized by the research team into major clinical groups for descriptive analysis.

Statistical analysis

Descriptive statistics were used to summarize participant characteristics and MSK outcomes. Categorical variables were reported as frequencies and percentages, and continuous variables as means with standard deviations. Incomplete questionnaires were reviewed prior to analysis. Available responses were included in descriptive analyses, and participants with sufficient data to determine MSK limitation status were retained in the primary analysis. Due to small sample size and limited cell counts, associations between MSK limitations and selected sociodemographic and health-related variables, including employment status, housing status, physical activity, age category, and presence of chronic medical conditions, were assessed using Fisher’s exact test. To aid interpretation of group-level burden, row-based proportion analyses were performed to compare the prevalence of MSK limitations within exposure categories. Statistical significance was defined as a two-sided p-value < 0.05. In addition, a multivariable logistic regression analysis was performed to evaluate factors associated with MSK limitations. Age, employment status, and presence of chronic medical conditions were selected a priori based on clinical relevance and prior literature. Adjusted odds ratios (OR) with 95% confidence intervals (CI) were reported.

## Results

A total of 62 participants were included in the analysis. 54 participants (87.1%) were male. Age distribution was skewed toward older adults, with 46 participants (74.2%) aged 50 years or older, of whom 27 (43.5%) were aged 60 years or above. Only 4 participants (6.4%) were younger than 40 years. Unemployment was common, with 38 participants (61.3%) reporting that they were currently unemployed, whereas 24 participants (38.7%) were employed. Housing instability was prevalent: 28 participants (45.2%) reported experiencing homelessness, 16 participants (25.8%) reported temporary housing (living with friends or family or residing in a shelter), and only 18 participants (29%) reported stable housing (Table 1).

**Table 1:** Demographics and socioeconomic characteristics of participants (N=62)

Demographics	Category	n	% (n/N)
Age	21-29	1	1.6
	30-39	3	4.8
	40-49	12	19.3
	50-59	19	30.6
	60+	27	43.5
Gender	Male	54	87.1
	Female	8	12.9
Employment Status	Employed	24	38.7
	Unemployed	38	61.3
Housing Status	Stable Housing	18	29.0
	Temporary Housing	16	25.8
	Homeless	28	45.2

General health and lifestyle characteristics

A total of 49 participants (79.0%) reported engaging in regular physical activity, while 31 participants (50.0%) reported a history of prior injuries resulting in impaired mobility. Use of assistive mobility devices, including canes, braces, or wheelchairs, was reported by nine participants (14.5%). Self-rated general health was most commonly described as fair by 23 participants (37.1%) or good by 21 participants (33.9%). Fourteen participants (22.6%) rated their general health as excellent, while four participants (6.4%) reported poor general health. A summary of health-related characteristics in the study population is shown in Table 2.

**Table 2:** Health-related characteristics of participants (N=62)

Variable	Category	n	% (n/N)
Physical Activity	Yes	49	79.0
	No	13	21.0
Previous Injuries Impairing Mobility	Yes	31	50.0
	No	31	50.0
Assisted Device Use	Yes	9	14.5
	No	53	85.5
General Health Perception	Excellent	14	22.6
	Good	21	33.9
	Fair	23	37.1
	Poor	4	6.4
Chronic Conditions	Yes	39	62.9
	No	23	37.1

Sleep duration varied across the cohort. 26 participants (42.0%) reported sleeping fewer than five hours per night, 16 participants (26%) reported sleeping five to six hours, 12 participants (19.3%) reported sleeping six to eight hours, and 8 participants (13.0%) reported sleeping more than eight hours per night.

MSK limitations by employment and housing status

**Figure 1:**
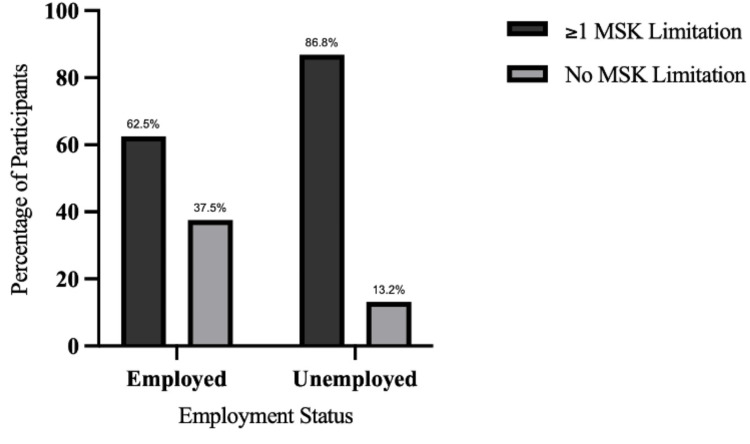
Prevalence of MSK limitations by employment status. The prevalence of MSK limitations differed significantly between unemployed and employed participants (p = 0.033)

The prevalence of MSK limitations differed by employment status (Figure 1). Among unemployed participants, 33 (86.8%) of 38 participants reported at least one MSK limitation, compared with 15 (62.5%) of 24 employed participants. Conversely, a greater proportion of employed participants reported no MSK limitations compared with unemployed participants (9 [37.5%] of 24 vs. 5 [13.2%] of 38). This difference was statistically significant (p = 0.033).

MSK limitations were also evaluated by housing status. Among participants experiencing homelessness, 22 (78.6%) of 28 participants reported at least one MSK limitation. Similarly, MSK limitations were reported by 13 (81.3%) of 16 participants living in temporary housing and by 13 (72.2%) of 18 participants with stable housing. Differences in the prevalence of MSK limitations across housing groups were not statistically significant (p = 0.739).

Health and functional characteristics

Musculoskeletal complaints were common among participants. UE difficulty, defined as problems using the arms for reaching, lifting, or carrying, was reported by 17 participants (27.4%), while UE pain during or after tasks was reported by 34 participants (54.8%). LE difficulty, including problems with walking, standing, or climbing stairs, was reported by 30 participants (48.4%), and LE pain was reported by 43 participants (69.4%). Back or neck pain during daily activities was reported by 40 participants (64.5%). Balance or coordination difficulties were noted by 31 participants (50%). Functional consequences were also frequently reported. 31 participants (50%) stated that they had modified their daily routine due to physical discomfort or limitation. Use of assistive devices, including canes or braces, was reported by 9 participants (14.5%). Mean UE strength was 3.85 ± 1.32, and mean LE strength was 3.94 ± 1.20 on a 5-point scale (1 = very weak; 5 = very strong). The mean posture rating was 3.50 ± 1.30, and satisfaction with the ability to perform daily activities averaged 3.79 ± 1.23.

Participants were also asked how frequently they experienced pain or discomfort during daily tasks. Responses were widely distributed: 15 participants (24.2%) reported pain “sometimes,” 9 participants (14.5%) “often,” and 12 participants (19.4%) “always.” Only 13 participants (21.0%) reported “never” experiencing pain.

Chronic conditions and MSK limitations

Chronic medical conditions were commonly reported in the study population. Overall, 39 (62.9%) of 62 participants reported having at least one chronic condition (Table 3). The most frequently reported conditions were metabolic disorders, including 21 participants (53.8%) with type 2 diabetes mellitus, 17 participants (43.6%) with cardiovascular conditions such as hypertension, and musculoskeletal diagnoses, including osteoarthritis in 9 participants (23.1%).

**Table 3:** Distribution of self-reported chronic health conditions by category among participants with chronic conditions (N = 39) † PVD includes arterial and venous insufficiency. § Dyslipidemia includes hypercholesterolemia and hyperlipidemia. ‡ CKD includes nephrotic glomerulopathy. ¶ Diagnoses not reported by participants were categorized as unspecified. Diagnoses were self-reported by participants and categorized by the research team for analysis. MSK: musculoskeletal; HTN: hypertension; T2DM: type 2 diabetes mellitus; PVD: peripheral vascular disease; CKD: chronic kidney disease; GAD: generalized anxiety disorder; ADHD: attention-deficit/hyperactivity disorder.

Category	Diagnosis	n	% (n/N)
Cardiovascular Conditions	HTN	17	43.6
	PVD †	6	15.4
	Arrhythmias, unspecified	1	2.6
Metabolic Conditions	T2DM	21	53.8
	Dyslipidemia §	4	10.3
Musculoskeletal Conditions	Osteoarthritis	9	23.1
	Osteoporosis	1	2.6
	Fibromyalgia	1	2.6
	Gout	1	2.6
	Disc Herniation	1	2.6
	Scoliosis	2	5.1
	Plantar Fasciitis	1	2.6
	Achilles Tendonitis	1	2.6
	Carpal Tunnel Syndrome	1	2.6
Neurologic Conditions	Peripheral Neuropathy	3	7.7
	Migraine Headaches	1	2.6
Renal Disease	CKD ‡	3	7.7
Mental Health Conditions	Depression	3	7.7
	GAD	2	5.1
	Psychosis	1	2.6
	ADHD	1	2.6
	Unspecified	1	2.6
Hematologic Conditions	Anemia	2	5.1
Others	Diverticulosis	1	2.6
	Hypoglycemia	1	2.6
	Cramps	1	2.6
	Unspecified ¶	1	2.6

**Figure 2:**
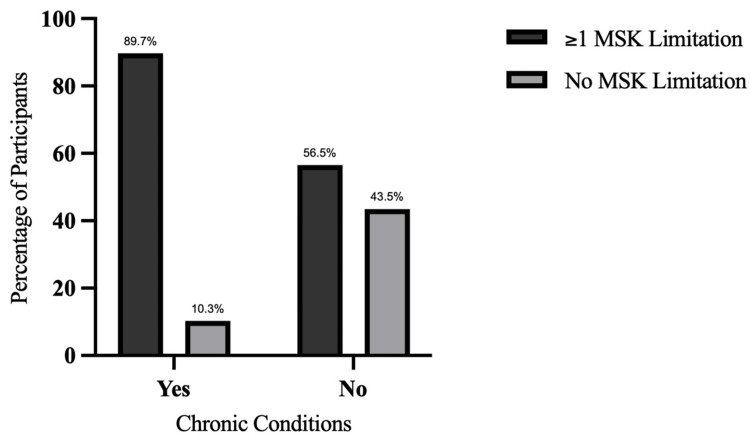
Prevalence of MSK limitations among participants with and without chronic conditions. MSK limitations were more prevalent among participants with chronic conditions compared with those without Statistical analysis was performed using Fisher’s exact test (p = 0.0042).

The prevalence of MSK limitations differed significantly by chronic disease status (Figure 2). Among participants with at least one chronic condition, 35 (89.7%) of 39 participants reported one or more MSK limitations, compared with 13 (56.5%) of 23 participants without chronic conditions reported MSK limitations. This difference was statistically significant (p = 0.0042).

Distribution and anatomical location of MSK limitations

Overall, 48 (77.4%) of 62 participants reported at least one musculoskeletal (MSK) limitation involving the UE, LE, or spine/back. Among affected participants, 18 (29.0%) reported a single MSK limitation, 17 (27.4%) reported limitations in two body regions, and 13 (21.0%) reported limitations involving all three regions. By anatomical region, limitations involving the spine or back were most frequently reported, affecting 40 participants (64.5%), followed by LE limitations in 30 participants (48.4%) and UE limitations in 17 participants (27.4%). The distribution demonstrates a predominance of axial and LE involvement within this underserved population.

Multivariable analysis

In multivariable logistic regression analysis, the presence of chronic medical conditions was associated with increased odds of MSK limitations after adjustment (OR = 7.76, 95% CI: 1.73-34.76, p = 0.007) (Table 4). Unemployment was also associated with higher odds of MSK limitations after adjustment (OR = 4.08, 95% CI: 1.01-16.38, p = 0.048). Age ≥50 years was not independently associated with MSK limitations after adjustment (OR = 5.78, 95% CI: 0.86-39.01, p = 0.072).

**Table 4:** Multivariable logistic regression analysis MSK: musculoskeletal; OR: odds ratio; CI: confidence interval

Variable	OR	95% CI	p-value
Chronic conditions	7.76	1.73-34.76	0.007
Unemployed	4.08	1.01-16.38	0.048
Age ≥ 50	5.78	0.86-39.01	0.072

## Discussion

This cross-sectional study of 62 patients attending an SRFC in Old San Juan underscores the substantial musculoskeletal (MSK) burden faced by underserved individuals in Puerto Rico. Nearly two-thirds of participants reported LE pain, and more than half reported back or neck pain, balance difficulties, or UE pain. These symptoms frequently resulted in activity modification and, in some cases, the use of assistive devices, highlighting the functional impact of MSK complaints in this population. Although most participants reported regular physical activity, this may reflect necessity-driven mobility, such as walking for transportation or access to services, rather than structured recreational exercise.

Our findings align with prior research demonstrating that MSK conditions are highly prevalent among people experiencing homelessness and other marginalized groups. In a Spanish shelter-based cohort, 98.4% of participants reported MSK pain of moderate intensity, with half experiencing pain at multiple sites [14]. Similarly, a scoping review of pain among individuals experiencing homelessness found that pain is often moderate to severe, frequently non-localized, and recurrent or persistent over time [15]. Although the prevalence of pain in our sample was somewhat lower, this difference may reflect variations in age distribution, chronic disease burden, or access to basic care through the free clinic setting.

The distribution of pain sites observed in our study mirrors patterns reported elsewhere. A systematic review of MSK injuries among homeless patients found that approximately 25% of injuries were MSK in nature, with high rates of arthritis, chronic pain, back strain, and foot-related conditions, particularly among older adults [16]. Foot pain and prior foot injuries were frequently reported, sometimes affecting more than half of respondents. Our finding that nearly two-thirds of participants experienced LE pain and half reported balance or coordination difficulties is consistent with these observations and underscores the importance of focused foot, gait, and mobility assessments in underserved clinical settings.

Participants in this study faced significant socioeconomic and health challenges. The majority were unemployed, many were unhoused or residing in shelters, and a substantial proportion reported sleeping fewer than five hours per night. These conditions are well-recognized contributors to poor MSK health, as unemployment, housing instability, and mental health stressors exacerbate pain, delay care-seeking, and limit access to rehabilitative services [2,4,7,8]. German health surveys of homeless individuals reported MSK disorders in 43% of participants and identified barriers to outpatient care, including lack of insurance, mistrust of healthcare systems, long travel distances, and competing survival priorities [5]. High rates of unmet medical needs, such as dental care, prescription medications, and surgical services, were also documented [5]. Together, these findings help explain why many individuals in our cohort delayed seeking care until symptoms may have become functionally limiting and emphasize the critical role of SRFC in addressing unmet MSK needs.

Chronic comorbidities were common in our cohort, with approximately 60% of participants reporting at least one chronic medical condition and nearly half reporting a prior MSK injury. Although our sample size limited the detection of statistically robust associations, the intersection of chronic disease and MSK impairment warrants particular attention. Prior studies among Puerto Rican adults have demonstrated high prevalences of obesity, diabetes, depressive symptoms, cardiovascular disease, and arthritis, all of which are associated with impaired physical function and mobility disability [10]. Data from the Boston Puerto Rican Health Study showed that obesity and depressive symptoms were significantly associated with mobility limitations, while poor balance was linked to obesity and histories of heart attack, stroke, and arthritis [10]. Additionally, arthritis prevalence among Puerto Rican adults aged 60-75 years was substantial, particularly among women, and depressive symptoms affected more than half of female participants [10]. These findings suggest that the chronic disease burden common in Puerto Rican communities may amplify MSK symptoms and contribute to functional decline.

These findings are further supported by multivariable analysis, in which chronic conditions showed the strongest association with MSK limitations, followed by unemployment. However, given the modest sample size and wide confidence intervals, these associations should be interpreted cautiously and as hypothesis-generating rather than causal.

Bone health represents another important but underexplored dimension of MSK vulnerability in this population. The Boston Puerto Rican Osteoporosis Study reported prevalences of osteoporosis and low bone mass of 10.5% and 43.3%, respectively, among adults aged 47-79 years, with age-adjusted osteoporosis prevalence notably higher in Puerto Rican men compared with non-Hispanic White and Mexican American men [12]. Although bone density was not assessed in our study, the high prevalence of LE pain, balance difficulties, and functional limitations observed may partially reflect underlying osteoporosis or osteoarthritis, highlighting the need for future studies that incorporate bone health assessment.

Our findings also underscore the psychosocial dimensions of MSK health. Nearly half of the participants rated their general health as fair or poor, and more than half reported significant sleep deprivation. Poor sleep quality and psychological distress are known to exacerbate pain perception and hinder recovery. A scoping review of pain among people experiencing homelessness noted that chronic pain frequently coexists with loneliness, depression, and psychological stress [15]. Similarly, follow-up studies of unhoused patients with inflammatory arthritis have shown higher morbidity and mortality, increased chronic disease burden, and persistent barriers to care, prompting calls for closer collaboration between specialty services and street medicine programs. These insights reinforce the need to address MSK complaints within a broader framework that integrates medical, mental health, and social services.

Importantly, functional limitation in this population may be particularly consequential given the necessity-driven nature of physical activity. For individuals facing housing instability and unemployment, mobility is often required to access shelter, food, employment opportunities, and healthcare. As such, limitations involving the spine and LE may have disproportionate effects on daily functioning, independence, and survival. The observed association between unemployment and a higher prevalence of MSK limitations may reflect a potentially bidirectional relationship, whereby MSK impairment could limit work capacity, while unemployment may worsen disease burden through reduced access to care and rehabilitation. However, this interpretation remains speculative because the cross-sectional design cannot determine temporality or causality.

Implications for practice

SRFC may be well-positioned to identify and address MSK conditions in underserved populations. Based on these preliminary findings, incorporating standardized MSK and functional screening into routine clinic visits may help identify pain, mobility impairment, and potential rehabilitative needs. Evidence from shelter-based physical therapy interventions in Spain demonstrated that brief, individualized physical therapy sessions significantly improved pain and self-perceived health among people experiencing homelessness [14]. Although such programs remain scarce, these findings suggest that integrating physical therapy services directly into free clinics or shelter settings, particularly through flexible, walk-in models, may enhance patient engagement and functional outcomes. Street medicine programs may further support continuity of care; prior studies have shown substantially higher short-term primary care engagement among unhoused individuals supported by street medicine compared with traditional referral pathways.

Limitations

This study has several limitations. The sample size was modest and derived from a single SRFC, which may limit generalizability to other settings. The study population was also predominantly male, older, and characterized by high rates of unemployment and housing instability; therefore, findings may not fully represent women, younger adults, or underserved populations in other regions of Puerto Rico. Outcomes were based on self-reported data and may be subject to recall bias, health literacy differences, and response bias. Chronic medical conditions were also self-reported and were not verified through medical records, which may introduce misclassification. The questionnaire was developed for local use and has not yet undergone formal psychometric validation; therefore, future studies should validate this tool in larger SRFC and underserved populations. In addition, the number of eligible patients approached and the response rate were not recorded, which limits assessment of selection bias. Although multivariable regression was performed, the modest sample size and wide confidence intervals suggest that these findings should be interpreted cautiously. Multiple subgroup comparisons were also performed without adjustment for multiple testing. Finally, employment status and MSK limitations were assessed cross-sectionally, so the observed relationship should be interpreted as associative and potentially bidirectional rather than causal. Nonetheless, these findings provide meaningful preliminary insight into the musculoskeletal health burden within an underserved population.

## Conclusions

MSK complaints were common among adults attending an SRFC in Puerto Rico, with the greatest burden involving the spine and LE. This burden occurred in the context of pronounced socioeconomic vulnerability, unemployment, necessity-driven physical activity, poor sleep, prior injuries, and a high prevalence of chronic disease. Although some associations between demographic factors and MSK outcomes were limited by sample size, MSK limitations were more prevalent among individuals who were unemployed or reported chronic medical conditions. These preliminary, hypothesis-generating findings suggest the potential value of systematic MSK screening, integrated management strategies, and strengthened partnerships with physical therapy, street medicine, and social services. Future larger, multi-site studies are needed to confirm these associations, evaluate root causes of MSK pain, and determine whether targeted interventions improve outcomes among underserved populations.

## Appendices

**Table 5:** Orthopedic and musculoskeletal function questionnaire Spanish version The questionnaire used for data collection in this study was developed by the authors and administered in both Spanish and English. The Spanish version represents the original instrument, and the English version is a direct translation for accessibility purposes. Credits: Developed by the authors.

Section	Question	Options
Sección 1: Demografía	1. Edad	21–29; 30–39; 40–49; 50–59; 60+
	2. Género	Masculino; Femenino; No binario; Prefiero no contestar
	3. Ocupación	Ventas; Servicio de alimentos; Atención médica; Servicio al cliente; Trabajo manual; Transporte; Educación; Desempleado/a; Trabajador/a independiente; Otro
	4. ¿Participa en actividades físicas regularmente?	Sí; No
	5. Tipo de actividad física	Caminar; Correr; Levantamiento de pesas; Yoga/Pilates; Deportes; Otro
	6. ¿Tiene o ha tenido lesiones?	Sí; No
	7. Parte del cuerpo afectada	Extremidad superior; Extremidad inferior; Columna; Otro
	8. Marque dónde siente dolor	Diagrama corporal
Sección 2: Extremidad Superior	1. ¿Dificultad en actividades diarias?	Sí; No
	2. Acciones difíciles	Levantar sobre cabeza; Cargar bolsas; Alcanzar objetos; Escribir/teclado; Otro
	3. ¿Dolor post actividad?	Sí; No
	4. Fuerza en brazos (1–5)	1; 2; 3; 4; 5
Sección 3: Extremidad Inferior	1. ¿Dificultad al caminar/subir escaleras?	Sí; No
	2. Acciones difíciles	Caminar >10 min; Escaleras; Estar de pie; Correr; Otro
	3. ¿Dolor en caderas/rodillas/pies?	Sí; No
	4. Fuerza en piernas (1–5)	1; 2; 3; 4; 5
Sección 4: Columna	1. ¿Dolor espalda/cuello?	Sí; No
	2. ¿Cuándo ocurre?	Sentado; De pie; Actividad física; Mañana; Otro
	3. ¿Dificultad equilibrio/coordinación?	Sí; No
	4. Postura (1–5)	1; 2; 3; 4; 5
Sección 5: Función General	1. Frecuencia de dolor	Nunca; Rara vez; A veces; A menudo; Siempre
	2. ¿Ajustes en rutina?	Sí; No
	3. Satisfacción (1–5)	1; 2; 3; 4; 5
	4. Uso de dispositivos	Sí; No
	5. Especificar dispositivos	Respuesta abierta
Sección 6: Salud General	1. Estado de salud	Excelente; Bueno; Regular; Malo
	2. Horas de sueño	<5; 5–6; 6–8; >8
	3. Condición crónica	Sí; No
	4. Especificar condición	Respuesta abierta
	5. Situación de vivienda	Hogar estable; Con familia/amigos; Refugio; Calle; Otro

**Table 6:** Orthopedic and musculoskeletal function questionnaire English version

Section	Question	Options
Section 1: Demographics	1. Age	21–29; 30–39; 40–49; 50–59; 60+
	2. Gender	Male; Female; Non-binary; Prefer not to say
	3. Occupation	Retail; Food service; Healthcare; Customer service; Manual labor; Transportation; Education; Unemployed; Self-employed; Other
	4. Physical activity	Yes; No
	5. Type of activity	Walking; Running; Weightlifting; Yoga/Pilates; Sports; Other
	6. Injuries	Yes; No
	7. Body part affected	Upper limb; Lower limb; Spine; Other
	8. Pain location	Body diagram
Section 2: Upper Limb	1. Difficulty using arms	Yes; No
	2. Difficult actions	Overhead lifting; Carrying bags; Reaching; Writing/typing; Other
	3. Pain after tasks	Yes; No
	4. Arm strength (1–5)	1; 2; 3; 4; 5
Section 3: Lower Limb	1. Difficulty walking/stairs	Yes; No
	2. Difficult actions	Walking >10 min; Stairs; Standing; Running; Other
	3. Pain in lower limb	Yes; No
	4. Leg strength (1–5)	1; 2; 3; 4; 5
Section 4: Spine	1. Back/neck pain	Yes; No
	2. When pain occurs	Sitting; Standing; Activity; Morning; Other
	3. Balance issues	Yes; No
	4. Posture (1–5)	1; 2; 3; 4; 5
Section 5: General Function	1. Pain frequency	Never; Rarely; Sometimes; Often; Always
	2. Routine adjustment	Yes; No
	3. Satisfaction (1–5)	1; 2; 3; 4; 5
	4. Use of aids	Yes; No
	5. Specify aids	Open response
Section 6: Health & Lifestyle	1. General health	Excellent; Good; Fair; Poor
	2. Sleep hours	<5; 5–6; 6–8; >8
	3. Chronic condition	Yes; No
	4. Specify condition	Open response
	5. Housing situation	Stable home; With family/friends; Shelter; Street; Other
